# Carbon-11 and Fluorine-18 Labeled Amino Acid Tracers for Positron Emission Tomography Imaging of Tumors

**DOI:** 10.3389/fchem.2017.00124

**Published:** 2018-01-15

**Authors:** Aixia Sun, Xiang Liu, Ganghua Tang

**Affiliations:** ^1^Guangdong Engineering Research Center for Translational Application of Medical Radiopharmaceuticals and Department of Nuclear Medicine, The First Affiliated Hospital, Sun Yat-sen University, Guangzhou, China; ^2^Department of Anesthesiology, The Sixth Affiliated Hospital, Sun Yat-sen University, Guangzhou, China

**Keywords:** positron-emitting AAs, carbon-11, fluorine-18, positron emission tomography, imaging, tumors

## Abstract

Tumor cells have an increased nutritional demand for amino acids (AAs) to satisfy their rapid proliferation. Positron-emitting nuclide labeled AAs are interesting probes and are of great importance for imaging tumors using positron emission tomography (PET). Carbon-11 and fluorine-18 labeled AAs include the [1-^11^C] AAs, labeling alpha-C- AAs, the branched-chain of AAs and *N*-substituted carbon-11 labeled AAs. These tracers target protein synthesis or amino acid (AA) transport, and their uptake mechanism mainly involves AA transport. AA PET tracers have been widely used in clinical settings to image brain tumors, neuroendocrine tumors, prostate cancer, breast cancer, non-small cell lung cancer (NSCLC) and hepatocellular carcinoma. This review focuses on the fundamental concepts and the uptake mechanism of AAs, AA PET tracers and their clinical applications.

## Introdution

Positron emission tomography (PET) can provide noninvasive molecular, functional and metabolic information. Thus, it is playing an increasingly important role in the diagnosis and staging of tumors, image-guided therapy planning, and treatment monitoring. 2-^18^F-fluoro-2-deoxy-D-glucose (^18^F-FDG) is a commonly used tracer for PET imaging. Based on the increased rate of glucose transport and glycolysis, the uptake of ^18^F-FDG in tumors cells is greater than that in normal cells. ^18^F-FDG has provided valuable information about tumors diagnosing, staging, and prognosis after surgery and therapy, but it has some limitations. On the one hand, due to the high uptake of ^18^F-FDG in the normal brain, it is difficult to obtain images with adequate contrast compared to primary or metastatic brain tumors (Zhao et al., 2014). On the other hand, some tumors, such as neuroendocrine tumors, renal cell carcinoma, prostate cancer and hepatocellular carcinoma, show low or nonspecific uptake, which may lead to false negative or positive results (Powles et al., 2007; Rioja et al., 2010; Bouchelouche and Choyke, 2015). Additionally, ^18^F-FDG PET is ambiguous for differentiating tumor from inflammation (Rau et al., 2002; Tang et al., 2003).

Besides glucose, certain AAs also serve as increasing energy sources and anabolic precursors for tumors. Positron nuclide-labeled AA tracers can overcome limitations of ^18^F-FDG for tumors imaging, and give information about AA metabolism in tumor. The uptake of AA PET tracers in the normal brain is significantly less than that of ^18^F-FDG, but the uptake of them in tumor is high. Thus, images with adequate contrast can be obtained using AA PET tracers for primary and metastatic brain tumors. Also, some AA PET tracers have an advantage over ^18^F-FDG in the differentiation of tumor from inflammation (Rau et al., 2002; Tang et al., 2003; Stober et al., 2006). It was reported that O-(2-^18^F-fluoroethyl)-L-tyrosine (^18^F-FET) and (S-^11^C-methyl)-L-methionine (^11^C-MET) have a significantly higher uptake in tumor cells than that in inflammatory cells. This different appearance can be contributed to major AAs transporter system L (Stober et al., 2006). They can also differentiate recurrent brain tumors from pseudo-progression or radiation necrosis among patients after surgery and radiotherapy (Niyazi et al., 2012; Galldiks et al., 2015a,b). In addition, some AA PET tracers with relatively little renal excretion can accurately detect prostate cancer and show high specificity and sensitivity, superior to ^18^F-FDG (Toth et al., 2005; Jana and Blaufox, 2006). Last, ^18^F-FDG is a nonspecific substrate for neuroendocrine tumors, but a few AA PET tracers are substrates of the enzyme aromatic AA decarboxylase (AADC), which are specific for neuroendocrine tumors imaging, such as 3,4-dihydroxy-6-^18^F-fluoro-L-phenylalanine (^18^F-FDOPA) and 5-hydroxy-L-[β-^11^C] tryptophan (^11^C-HTP) (Jager et al., 2008; Oberg and Castellano, 2011). This review focuses on the fundamental concepts of AAs and the uptake mechanism of AAs, AA PET tracers and their clinical applications.

## Fundamental concepts and uptake mechanisms of AAs

L-AAs, as essential small-molecule nutrient substances, are crucial for maintaining cell growth and nitrogen balance. Their biological functions are involved in metabolism, protein synthesis, cell signaling transduction, regulating gene expression. They are also precursors for the synthesis of hormones, neurotransmitter, and nitrogenous substances. L-AAs are commonly found in proteins and are either obtained from intracellular protein recycling or are transported into the cell from the extracellular surroundings (Stryer, 1995).

**Figure 1:**
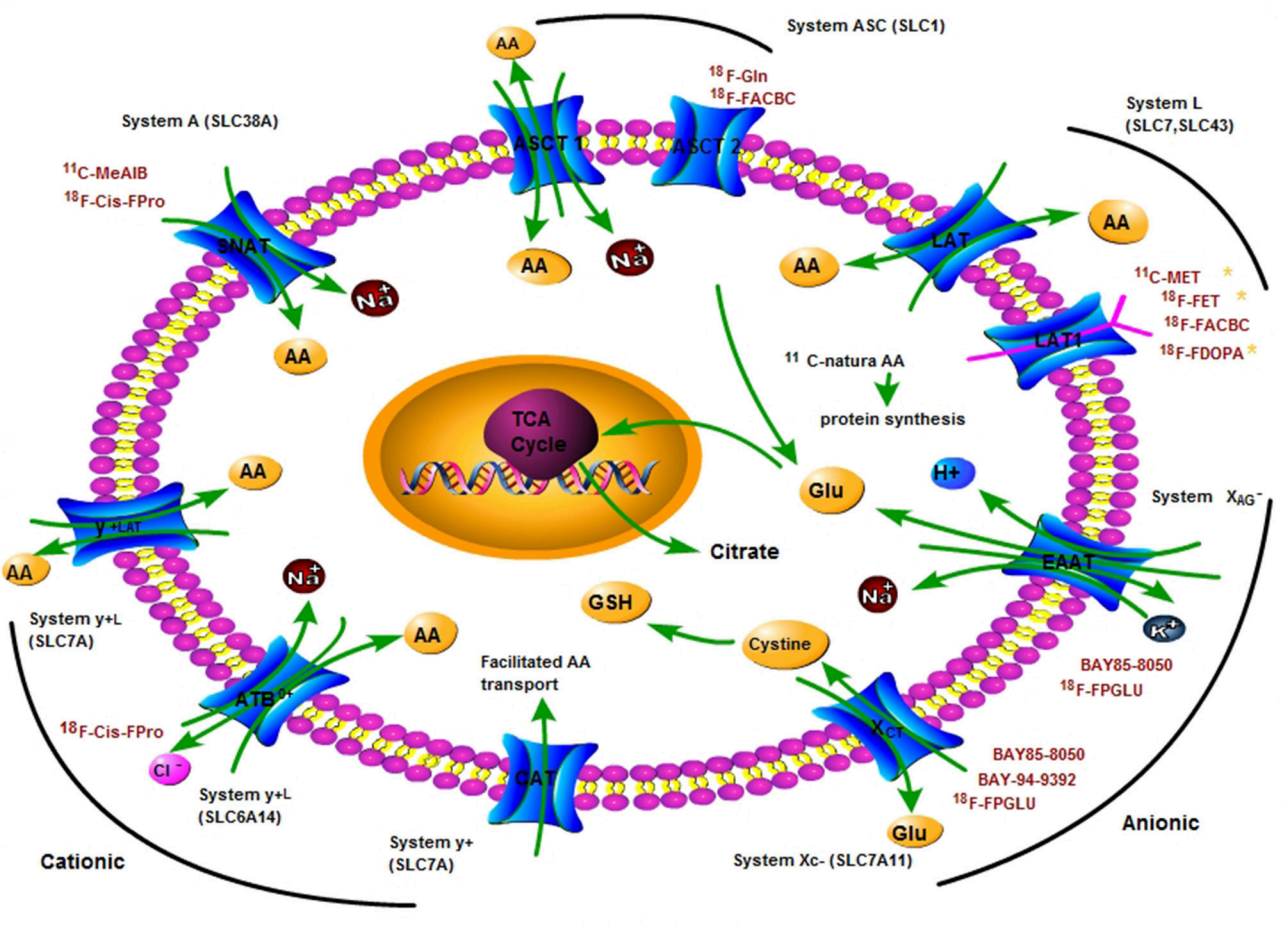
A principle scheme of the metabolic pathways and substrates accounting for the intracellular uptake of key clinical amino acids PET tracers for imaging tumor metabolism. Positron nuclide-labeled amino acids are shown in red colored words. AA, amino acid; ASCT, L-alanine, L-serine, cysteine transporter; ASCT2, ASC-type amino acid transporter 2 (SLC1A5); Gln, glutamine; Glu, glutamate; LAT1, L-type amino acid transporter 1 (SLC7A5); SNAT, system A amino acid transporter; EAAT, Excitatory amino acid transporters; xCT, a light chain of anionic amino acid transporter system X_C_− (SLC7A11); TCA, tricarboxylic acid cycle.

The transporters mediate AA transport across plasma membranes in mammals and are divided into several “systems.” The systems present various transporting mechanisms, including dependence on sodium and independence on sodium, tissue expression patterns, substrate specificity and sensitivity to pH or hormones (Utsunomiya-Tate et al., 1996; Castagna et al., 1997). Cells possess different transport systems in their plasma membranes, consisting of generally existed transport systems (such as systems A, ASC, L, y^+^ and X_AG_−, X_C_−), and tissue-specific transport systems (such as systems B^0^, and b^0, +^) (Palacin et al., 1998). Here, we focus on describing their general features and transport mechanism of AAs, as shown in Table 1 and Figure 1.

**Table 1:** Summary of AA transporters.

**Transporter**	**Gene name**	**System and mechanism of transport**	**Substrate**	**Inhibitors/blockers**
SNAT1	*SLC38A1*	Na^+^-dependent system A, concentrative	Small neutral AAs	MeAIB
SNAT2	*SLC38A2*			
SNAT4	*SLC38A4*			
ASCT1	*SLC1A4*	Na^+^-dependent system ASC, exchange		
ASCT2	^*^SLC1A5		L-Ala, L-Cys, L-Gln, L-Ser, L-Thr	L-γ-glutamyl-p-nitroanilide (GPNA) Esslinger et al., 2005; Schulte et al., 2015 Benzylserine Jager et al., 2008, Glupnitroanilide Bhutia et al., 2015
GLYT1, GLYT2	*SLC6*	Na^+^-dependent system G	Gly, Sar	
SN1, SN2	*SLC38*	Na^+^-dependent system N, concentrative	Gln, Asn, His	
Taut	*SLC6*	Na^+^-dependent β-system	β-Ala, Tau	
LAT1	^*^SLC7A5	Na^+^-independent system L, Exchange, heterodimer with 4F2hc	Large neutral L-AAs	BCH Rosilio et al., 2015
LAT2	*SLC7A8*			
LAT3	*SLC43A1*	Na^+^-independent system L, Facilitated		BCH, *N*-ethylmaleimide Ogihara et al., 2015
LAT4	*SLC43A2*			
Asc-1 Asc-2	*SLC7*	Na^+^-independent system asc	Ala, Ser, Thr, Cys	
TAT1	*SLC16*	Na^+^-independent system T	Aromatic AAs	
ATB^0, +^	^*^SLC6A14	System B^0, +^,Na^+^ and Cl^−^, concentrative	Neutral and basic AAs	α-Methyl-L-Trp Bhutia et al., 2015
CAT-1	*SLC7A1*	Na^+^-independent system y^+^, Facilitated	Lysine, histidine, arginine	*N*-ethylmaleimide Nel et al., 2012
CAT-2A/2B	*SLC7A2*			
CAT-3	*SLC7A3*			
y^+^LAT1	*SLC7A7*	Na^+^-independent system y^+^L,exchange heterodimer with 4F2hc	Cationic, large neutral AAs	BCH selective inhibitor
y^+^LAT2	*SLC7A6*			
BAT1/b0, +AT•rBAT	*SLC7*	System b^0, +^, Exchange, heterodimer with D2/rBAT/NBAT	Cationic, large neutral AAs	BCH
EAAT1	*SLC1A1*	System X_AG_−, Na^+^ cotransport and K^+^ counter transport	Glutamate, aspartate	The phorbol ester 12-myristate 13-acetate (TPA, 0-1000 nM) Pan et al., 1995a
GLT-1(EAAT2)	*SLC1A2*			
GLAST (EAAT3)	*SLC1A3*			
EAAT4	*SLC1A6*			
EAAT5	*SLC1A7*			
xCT	^*^SLC7A11	System X_C_−, Na^+^-independent, but Cl^−^ dependent glutamate/cysteine exchange, heterodimer with4F2hc	Glutamate/cystine	Sulfasalazine, Erastin, Sorafenib (S)-4-Carboxyphenyl glycine Bhutia et al., 2015 L-a-aminoadipate Lewerenz et al., 2013

* *Low-level expression in normal tissues, but up-regulated expression in many human tumors*.

*AAs, amino acids; MeAIB, N-methyl aminoisobutyric acid; BCH, 2-amino-endo-bicyclo[2,2,1]heptane-2-carboxylic acid*.

System A is Na^+^-dependent transporter for serving mainly small aliphatic AAs, such as serine, alanine, and glutamine. It is a member of the solute carrier 38 (*SLC38*) gene family. Three subtypes of system A have been isolated: sodium-coupled neutral AA transporter 1 (SNAT1), 2, and 4. SNAT 3 and 5 belong to the system N (glutamine preferring) AA transport family, which is also a member of the *SLC38* gene family (Broer, 2014). System A and system N are all directly concentrative and function essentially with a monodirectional efflux. System A transports AAs with the *N*-methyl group and *N*-methyl aminoisobutyric acid (MeAIB) is a specific inhibitor that can inhibit system A transport activity due to competitive saturation effects. Meanwhile, the activity of transporters is affected by many factors (Shotwell et al., 1983). The activity of system A is sensitive to pH alterations, highly down-regulated by acidic extracellular surroundings, and up-regulated by glucagon, insulin, and growth factors (Castagna et al., 1997).

The ASC system is Na^+^-dependent exchanger capable of mediating net influx or efflux, with substrates (L-alanine, L-serine, L-cysteine, and L-glutamine) and a member of solute the carrier family 1(*SLC1*) (Castagna et al., 1997). Two subtypes have been isolated: ASC-Type AA transporter 1 (ASCT1) and ASC-Type AA transporter 2 (ASCT2). ASCT2 utilizes an intracellular gradient of AAs, efflux of intracellular AAs in exchange for extracellular AAs. Glutamine is a key substrate of ASCT2 with important roles in tumor metabolism (Fuchs et al., 2007). ASCT2 is over-expressed in many human tumor cell lines including hepatocellular carcinoma, prostate, breast, glioma, and colon tumor cell lines (Li et al., 2003; Fuchs and Bode, 2005). L-γ-glutamyl-p-nitroanilide (GPNA) is used as a specific inhibitor of ASCT2 transporter activity (Schulte et al., 2015). The activity of system ASC is pH-insensitive within a range of pH 5.65–8.2 (Fuchs and Bode, 2005; Kanai et al., 2013).

The Na^+^-independent system L is the major route that takes up branched and aromatic AAs from the extracellular space, such as phenylalanine, isoleucine, tryptophan, valine, methionine and histidine (Castagna et al., 1997). Four subtypes of system L have been isolated: L-type AA transporters 1 (LAT1), LAT2, LAT3, and LAT4. LAT1 and LAT2 are members of the *SLC7* gene family, while LAT3 and LAT4 are members of the *SLC43* gene family. LAT1 and LAT2 possess “4F2 light chains” containing 12 putative membrane-spanning domains, which covalently bind a type-II membrane glycoprotein heavy chain (4F2hc) with disulfide bridges to produce a functional heterodimeric transporter. LAT3 and LAT4, without 4F2hc, facilitate the transport of AAs (Fuchs and Bode, 2005; Aiko et al., 2014). System L plays an important role for AAs crossing the placenta barrier and the blood-brain barrier (Christensen, 1990). 2-amino-2-norbornane-carboxylic acid (BCH) is a specific inhibitor for system L transporter activity (Palacin et al., 1998; Babu et al., 2003).

The cationic AA transporters include systems B^0, +^, y^+^, and y^+^L, and the anionic AA transporters contain systems X_AG_− and X_C_−. Systems B, B^0^, B^0, +^ y^+^, and y^+^L are related Na^+^-dependent transporter systems. They mediate the absorption of branched-chain, aliphatic and aromatic AAs. Systems B and B^0^ are tissue-specific transport systems and present in renal proximal tubular and intestinal epithelial brush-border membranes. Both systems are more broadly specific for neutral AAs than systems A and ASC. System y^+^ transporters are members of the *SLC7* gene family. Four subtypes, CAT-1, CAT-2 (A and B), CAT-3, and CAT-4, have been recognized from a subfamily of the *SLC7* gene family. CAT-1 is a exchanger targeting unessential AAs, and the action of CAT-4 remains unknown (Hammermann et al., 2001). System y^+^ transports cationic AAs and some neutral AAs, such as lysine and arginine, resulting in electrogenic transport (Castagna et al., 1997; Palacin et al., 1998). System y^+^L transporters are members of the SLC7 gene family as well. Two subtypes (y^+^LAT1 and y^+^LAT2) have been identified, and they create heterodimers with the 4F2hc glycoprotein to be functional AA transporters, such as the LAT1 and LAT2 transporters from system L. System y^+^L serves large neutral and cationic AAs with an exchange mechanism. ATB^0, +^ belongs to the *SLC6* gene family and serves cationic and neutral AAs in the presence of sodium and chloride. b^0, +^AT belongs to the *SLC7* gene family, which constitutes a functional heterodimer with the glycoprotein D2/rBAT/NBAT and serves cationic and neutral AAs via an exchange mechanism in the absence of sodium (Torrents et al., 1998; Hammermann et al., 2001).

System X_C_− is Na^+^-independent and Cl^−^-dependent heterodimeric AA transporter (Baker et al., 2002; Lewerenz et al., 2012, 2013), an obligate, electroneutral, cysteine/glutamate antiporter, exchanges extracellular cystine for intracellular glutamate (Lo et al., 2008; Lewerenz et al., 2012). It is composed of a subunit xCT light chain and a subunit 4F2 heavy chain (4F2hc). xCT is a member of *SLC7*, member 11 (SLC7A11), and phosphorylation of xCT can modulate the activity of system X_C_− (Baker et al., 2002; Lo et al., 2008; Lewerenz et al., 2012). It is not only a potential target for therapy but also a potential PET biomarker for imaging the system X_C_− activity of cancer and other diseases (Lo et al., 2008; Reissner and Kalivas, 2010; Koglin et al., 2011).

System X_AG_− is Na^+^-dependent and K^+^-dependent and transports acidic AAs, such as glutamate and aspartate (Dall'Asta et al., 1983; Pan et al., 1995b). Excitatory AA transporters EAAT1 (GLAST), EAAT2 (GLT-1), EAAT3 (EAAC1), and EAAT4 are members of the system X_AG_− AA transport family (Howell et al., 2001) and are neuronal/epithelial high affinity glutamate transporters (Yin et al., 2014). They are encoded by the *SLC1A1, SLC1A2, SLC1A3, SLC1A6, SLC1A7*, respectively (Kanai et al., 2013; Bianchi et al., 2014).

The transporter systems mentioned above are the main targets for AA metabolism PET imaging of tumors (Jager et al., 2001). Tumor cells utilize more AAs compared with normal cells to satisfy their rapid proliferation and invasion demands. And studies indicated that the expression of AA transporters is higher in tumor cells than that in normal tissue, especially LAT1, ASCT2, xCT, and ATB^0, +^ and so on (Karunakaran et al., 2011; Toyoda et al., 2014; Schulte et al., 2015). Both ASCT2 and LAT1 are upregulated three-fold in the most of cancerous tissues. LAT1 has been proven to be associated with tumor growth (Kaira et al., 2013), for example ^11^C-MET, ^18^F-FET, and ^18^F-FDOPA are the most widely used AA PET tracers for imaging brain tumors. System A and cationic or anionic AA transporters are over-expressed in dividing cells in certain human cancers (Bussolati et al., 1996). Many examples are showed in Table 2. Tumor cell accumulation of AA PET tracers mainly depends on the rate and mechanism of AAs transport. Based on the over-expression of AA transporters, the uptake of AA PET tracers in tumor cells is greater than that in normal cells (Mossine et al., 2016).

**Table 2:** Uptake mechanism and clinical application of important AA PET tracers for tumors imaging.

**Tracer**	**Labeling position**	**Mechanism and transporter**	**Application**
^11^C-Leu, ^11^C-Tyr, ^11^C-Phe	[1-^11^C] COOH	Protein synthesis	Brain tumors, *in vivo* protein synthesis rate
^11^C-AIB, ^11^C-Met		System A transport	Sarcoma, melanoma Lebarre et al., 1991; de Boer et al., 2003; Veronese et al., 2012; Nishii et al., 2013
^11^CH3-AIB	Labeled α-carbon	System A transport	Head and neck cancer
^11^CH3-AMT			Glioma Juhasz et al., 2011
^11^C-HTP, ^11^C-DOPA	Labeled branched-chain	System L transport	Neuroendocrine tumors Toumpanakis et al., 2014
^11^C-MET^*^		System L (LAT1) transport/protein synthesis	Brain tumors and prostate cancer Ceyssens et al., 2006; Jana and Blaufox, 2006
^11^C-MCYS		System L, ASC and B^0, +^ transport	Brain tumors Deng et al., 2011; Huang et al., 2015
^18^F-FDOPA^*^		System L (LAT1) transport	Brain tumors, neuroendocrine tumors
^18^F-OMFD		System L (LAT1) transport	Brain tumors Gulyas and Halldin, 2012
^18^F-FET^*^		System L transport	Brain tumors Mossine et al., 2016
^18^F-FMT		System L (LAT1) transport	Brain tumors
^18^F-FGln		System L transport	Brain tumors Gulyas and Halldin, 2012
8F-2S,4S-FSPG (BAY 94-9392)		System L and ASC transport	Hepatocellular carcinoma, in non-small cell lung cancer Chopra, 2004
BAY 85-8050		System X_C_− transport	Healthy volunteers Smolarz et al., 2013b
^18^F-FAMT		System X_C_− and X_AG_− transport	Head and neck cancer, lung cancer Miyakubo et al., 2007
^18^F-FACBC, ^18^F-FACPC		System L transport	Prostate cancer Schuster et al., 2011
^11^C-MeAIB	*N*-substituted labeled	System A transport	Head and neck cancer Sutinen et al., 2003
^18^F-Cis-FPro	Labeled branched-chain/*N*-substituted labeled	System A and system B^0+^ transport/protein synthesis	Head and neck cancer, pulmonary, and mediastinal mass Stoffels et al., 2008

* *The most widely used AAs PET tracers in clinical settings*.

## AA pet tracers

Most AA PET tracers are labeled with positron radionuclides ^11^C and ^18^F. Theoretically, almost all AAs be labeled with ^11^C, however, their short half-life (20 min, 100% of beta positron decay) is not suitable for delayed PET imaging. To overcome this shortcoming of ^11^C and to facilitate the utility of AA PET tracers in hospitals without on-site cyclotron and labeling equipment, a series of ^18^F labeled AAs (half-life of 110 min, 97% of beta positron decay) were developed (Mossine et al., 2016). Based on that AAs have a common molecular formula [R-CH-(NH_2_)-COOH], with a carboxylic acid group (-COOH), an amino group (-NH_2_) linking to the alpha-carbon atom (-CH-), and branched-chain group (-R). Thus, ^11^C and ^18^F labeled AAs are divided into [1-^11^C] AAs ([1-^11^C]AAs), alpha-C labeled AAs (alpha-C labeled AAs), labeled branched-chain AAs (branched-chain AAs), and *N*-substituted labeled AAs (*N*-substituted labeled AAs), which include natural and non-natural AAs.

Labeled natural AAs associated with structure-changed and structure-unchanged labeled AAs. Structure-unchanged labeled natural AAs, such as [1-^11^C] AAs and ^11^C-Met, do not chemically change the structure of AAs and can maintain the prototype structure and the fundamental pharmacodynamics and pharmacokinetics characteristics of AAs. So, they are mainly incorporated into protein synthesis, with minor AA transport. On the contrary, structure-changed labeled AAs (such as ^18^F-FET, (S-^11^C-methyl)-L-cysteine) do chemically change the structure of AAs, which are slightly incorporated into protein synthesis. Like structure-changed labeled AAs, labeled non-natural AAs (such as ^18^F-FDOPA, ^11^C-HTP) are mainly involved into AA transport. Most important ^11^C- and ^18^F-labeled AA tracers are shown in Table 2.

[1-^11^C]AAs have ^11^C-labeled at the alpha-carboxylate (-COOH) position, [1-^11^C]-labeled natural AAs such as L-[1-^11^C]-leucine (^11^C-Leu) (Veronese et al., 2012), L-[1-^11^C]tyrosine (^11^C-Tyr) (de Boer et al., 2003), L-[1-^11^C]phenylalanine (^11^C-Phe) (Lebarre et al., 1991) and L-[1-^11^C]methionine (^11^C-Met) (Ishiwata et al., 1988) are mainly incorporated into protein synthesis, and can be used to measure the rates of the protein synthesis. [1-^11^C]-labeled non-natural AAs, such as carboxyl-^11^C-1-α-aminoisobutyric acid (^11^C-AIB), carboxyl-^11^C-1-aminocyclopentanecarboxylic acid (^11^C-ACPC), and carboxyl-^11^C-1-aminocyclopentane carboxylic acid (^11^C-ACBC), etc., are not incorporated into protein synthesis and have been used for imaging of tumor AA transport in several studies (Washburn et al., 1978; De Vis et al., 1987).

Labeled alpha-carbon AAs have radiolabeled at alpha-carbon (-CH-) position of AAs, which are rarely reported. α-[^11^C-methyl]-L-tryptophan (^11^C-AMT) and α-[^11^C-methyl]-aminoisobutyric acid (^11^CH_3_-AIB) are typical examples that have been used for tumors imaging by measuring the rate of AA transport (Juhasz et al., 2011).

Labeled branched-chain AAs have radiolabeled at branched-chain group (-R) of AAs. Labeled branched-chain natural AAs with unchanged structure are rare, for example (S-[^11^C]methyl)-L-methionine (^11^C-MET). Most labeled branched-chain natural AAs are changed into different structure labeled AAs from natural AAs, such as ^18^F-FET, 2-^18^F-fluoro-L-tyrosine (2-FTYR), 6-^18^F-L-m-tyrosine (^18^F-FMT), O-(3-^18^F-fluoropropyl)-L-tyrosine (^18^F-FPT), 2-^18^F-L-phenylalanine, cis-^18^F-fluoroproline (cis-Fpro), (4S)-4-(3-^18^F-fluoropropyl)-L-glutamate (BAY 94-9392,^18^F-FSPG), (2S,4R)-4-^18^F-L-glutamate (BAY85-8050, 4F-GLU), L-(5-^11^C)-glutamine, (2S,4R)-4-^18^F-L-glutamine (^18^F-(2*S*,4*R*)4F-GLN), (2S,4S)-4-(3-^18^F-fluoropropyl) glutamine (^18^F-FPGln), and (S-^11^C-methyl)-L-cysteine (^11^C-MCYS) (Deng et al., 2011; Huang et al., 2015). Labeled branched-chain non-natural AAs include labeled branched-chain D-AAs and labeled branched-chain L-non-natural AAs. The former includes D-^11^C-fluoromethyltyrosine, D-^18^F-fluoromethyltyrosine (^18^F-D-FMT) (Burger et al., 2014) and (S-^11^C-methyl)-D-cysteine (^11^C-DMCYS) (Huang et al., 2015). The latter includes 3-^18^F-α-methyltyrosine (^18^F-FAMT), 1-amino-3-^18^F-fluorocyclobutane-1-carboxylic acid (^18^F-FACBC), 3-*O*-methyl-6-^18^F-L-3, 4-dihydroxyphenylalanine (^18^F-OMFD), (S)-2-amino-3-[1-(2-^18^F-fluoroethyl)-1H-[1,2,3]triazol-4-yl]propanoic acid (^18^F-AFETP), 3-^18^F-2-methyl-2-(methylamino)propanoic acid (^18^F-MeFAMP), 3,4-dihydroxy-6-^18^F-L-phenylalanine (^18^F-FDOPA), anti-1-amino-2-^18^F-fluorocyclopentane-1-carboxylic acid (anti-2-^18^F-FACPC), 5-^18^F-L-aminosuberic acid (^18^F-FASu), ^11^C-HTP, L-[β-^11^C]DOPA (^11^C-DOPA), L-[β-^11^C] dopamine, and ^18^F-fluoropropyl-L-tryptophan (^18^F-FPTP) (Jager et al., 2001; McConathy and Goodman, 2008; McConathy et al., 2012; He et al., 2013; Huang and McConathy, 2013b; Webster et al., 2014). Among these, ^18^F-FAMT, ^18^F-FET, ^18^F-D-FMT, 2-FTYR, ^18^F-FDOPA, ^18^F-FMT, ^18^F-Cis-FPro, ^18^F-OMFD, ^18^F-FACBC, ^18^F-FACPC, ^11^C-HTP, ^11^C-DOPA, BAY 94-9392, BAY85-8050 and ^18^F-(2*S*, 4*R*)4F-GLN have been used in clinical PET imaging of tumors. Most of labeled branched-chain non-natural AAs are involved in AA transport and a few are incorporated into protein synthesis. However, 2-FTYR and ^18^F-Cis-Fpro are involved in AA transport and protein synthesis (Jager et al., 2001; Laverman et al., 2002).

*N*-substituted labeled AAs have radiolabeled at -NH_2_ group of AAs. α-[*N*-methyl-^11^C]-methylaminoisobutyric acid (^11^C-MeAIB) and α-(*N*-[1-^11^C]acetyl)-aminoisobutyric acid (Prenant et al., 1996) are *N*-substituted labeled non-natural AAs targeting transport system A. ^11^C-MeAIB has been used for clinical PET imaging of tumor (Sutinen et al., 2003). Although several *N*-substituted labeled natural AAs, such as p-^18^F-fluorohippurate (^18^F-PFH) as a glycine analog, have been reported, their transport mechanisms remain unknown (Awasthi et al., 2011). *N*-substituted labeled natural AAs targeting different AA transport systems, such as *N*-(2-^18^F-fluoropropionyl)-L-methionine (^18^F-FPMET), *N*-(2-^18^F-fluoropropionyl)-L-glutamic acid (^18^F-FPGLU), *N*-(2-^11^C-methyl)-L-glutamic acid (^11^C-MGLU), were first reported by our research group (Hu et al., 2013, 2014). ^18^F-FPGLU is a potential AA PET tracer for tumor imaging and can be used for clinical tumor imaging in the near future. Our studies showed that ^18^F-FPGLU is mainly transported via X_AG_− and X_C_− (shown in Figure 1) (Hu et al., 2014; Tang et al., 2015).

## Clinical applications

**Figure 2:**
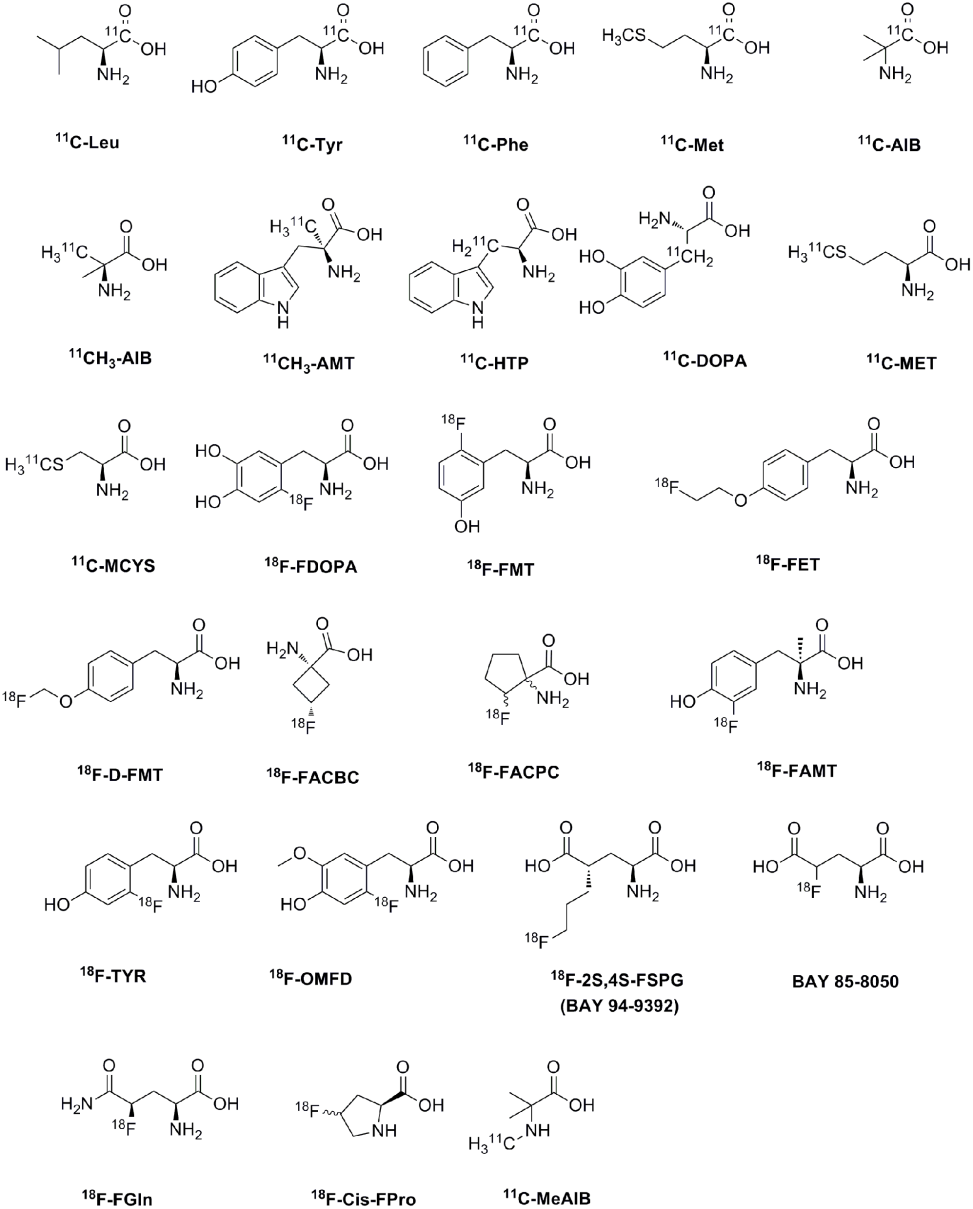
The chemical formula of amino acid PET tracers commonly used for clinical tumor imaging.

AA PET tracers were first used to measure the rate of protein synthesis *in vivo* (Vaalburg et al., 1992; Ishiwata et al., 1993; Paans et al., 1996). For example, ^11^C-labeled natural AAs, such as L-leucine, L-methionine, L-phenylalanine and L-tyrosine, are used to measure the protein synthesis rate since they incorporate into proteins or wash out with decarboxylation and oxidation (Ishiwata et al., 1996; Langen et al., 2006). Nowadays, AA transports seem to be more important than protein synthesis for the imaging of tumor metabolism *in vivo* (Ploessl et al., 2012; Lewis et al., 2015). A wide range of ^11^C and ^18^F AAs have been developed as PET tracers for clinical tumor imaging, as shown in Table 2 and Figure 2. The established AA tracers are used for imaging of brain tumors, neuroendocrine tumors, and prostate cancer, and other tumors.

### Brain tumor

Though ^18^F-FDG has been used in PET imaging of brain tumors, there exists weaknesses as mentioned (Olivero et al., 1995; Suchorska et al., 2014; Zhao et al., 2014; Tomura et al., 2015). AA PET tracers can overcome its limitations and provide a better description of tumor boundaries, which is important for surgical interventions, targeting biopsies, and radiation therapy (Suchorska et al., 2014). And ^18^F-FDG has been replaced by AA PET tracers or its analogs in clinical settings. The most widely used AA PET tracers are ^11^C-MET, ^18^F-FET, and ^18^F-FDOPA (Gulyas and Halldin, 2012; Wang et al., 2014).

**Figure 3:**
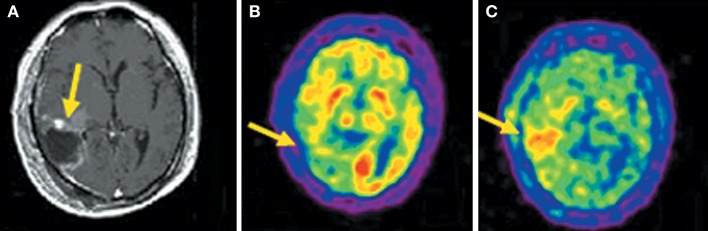
Images (Axial) of a 45-year-old man with a history of attempted resection of World Health Organization (WHO) grade glioma. **(A)** Subsequent new abnormal enhancing lesion (arrow) on Magnetic resonance (MRI). **(B)** ^18^F-FDG PET imaging illustrated patching-shaped hypormetabolism in the right temporal lobe (arrow). **(C)** ^11^C-MCYS PET imaging showed a patching-shaped hyperrmetabolism lesion (arrow), which was predominant high-grade tumor recurrence confirmed on histopathology. This figure is reproduced with permission from Deng et al. (2011), Figure 5 © by the Society of Journal of Nuclear Medicine Imaging, Inc.

Compared to ^18^F-FDG, the superior diagnostic accuracy of ^11^C-MET has been demonstrated in detecting, grading, delineating and searching recurrences, prediction of prognosis and evaluation of response to treatment (Nariai et al., 2005; Van Laere et al., 2005; Ceyssens et al., 2006; Galldiks et al., 2006). However, the sensitivity of ^11^C-MET was lower in the studies with high proportions of low-grade glioma (Hatakeyama et al., 2008; Glaudemans et al., 2013), which is the most universal type of primary brain tumor. Moreover, there is not yet enough evidence about grading glioma, and its use in differentiating tumor recurrences from radiation necrosis is controversial (Ishii et al., 1993; Sonoda et al., 1998; Nakagawa et al., 2002; Tsuyuguchi et al., 2004; Minamimoto et al., 2015). ^11^C-MCYS, a new AA PET tracer for tumor imaging, is reported that it, as analog of ^11^C-MET, appeared to have potential value as a tumor PET-imaging tracer (Figure 3) (Deng et al., 2011; Huang et al., 2015).

**Figure 4:**
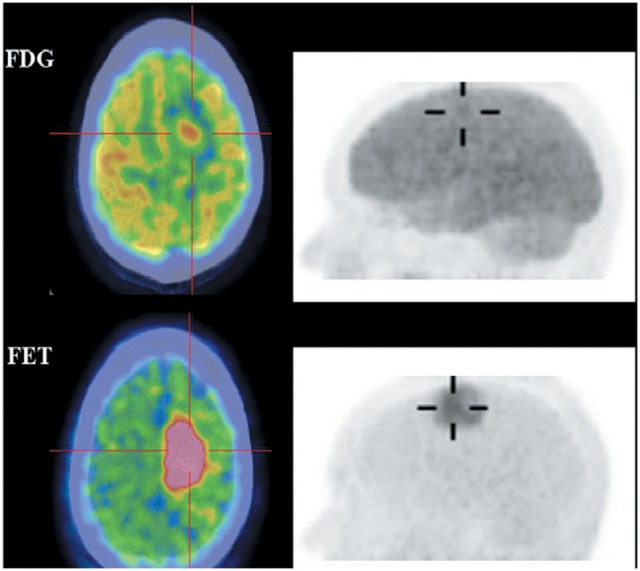
Images of a patient with recurrent glioma of World Health Organization (WHO) grade II oligodendrocytoma histologyon the background of WHO grade III anaplastic astrocytoma on initial diagnosis. Axial ^18^F-FDG (top), ^18^F-FET (bottom) fused PET/CT (left) and lateral maximum intensity projection images (right). ^18^F-FET imaging illustrated that the recurrent tumor in the right frontal lobe (cross-hairs) was better visualized and defined, and had a much lower brain uptake background to allow a good tumor-background contrast. This figure is reproduced with permission from Lau et al. (2010), Figure 4 © by the Society of Journal of Clinical Neuroscience, Inc.

^18^F-FET and ^18^F-FDOPA are derivatives of ^18^F-labeled L-phenylalanine and L-tyrosine, which target system L transporters to detect brain tumors. ^18^F-FET provides both good-contrast PET images of brain tumors (Figure 4) (Langen et al., 2006; Lau et al., 2010; Dunet et al., 2012) and valuable information about differentiating low-grade from high-grade tumor (Popperl et al., 2007; Dunet et al., 2012; Jansen et al., 2015). Dynamic ^18^F-FET examinations show high diagnostic accuracy in patients with suspected tumor progression or recurrence in clinical settings (Lau et al., 2010; Dunet et al., 2012). ^18^F-FET also can differentiate recurrent brain tumor from pseudoprogression and radiation necrosis (Niyazi et al., 2012; Galldiks et al., 2015a,b). Additionally, ^18^F-FET has a lower uptake by inflammatory cells than ^11^C-MET or ^18^F-FDG and it clearly delineates tumors from inflammation (Gulyas and Halldin, 2012; Nedergaard et al., 2014).

**Figure 5:**
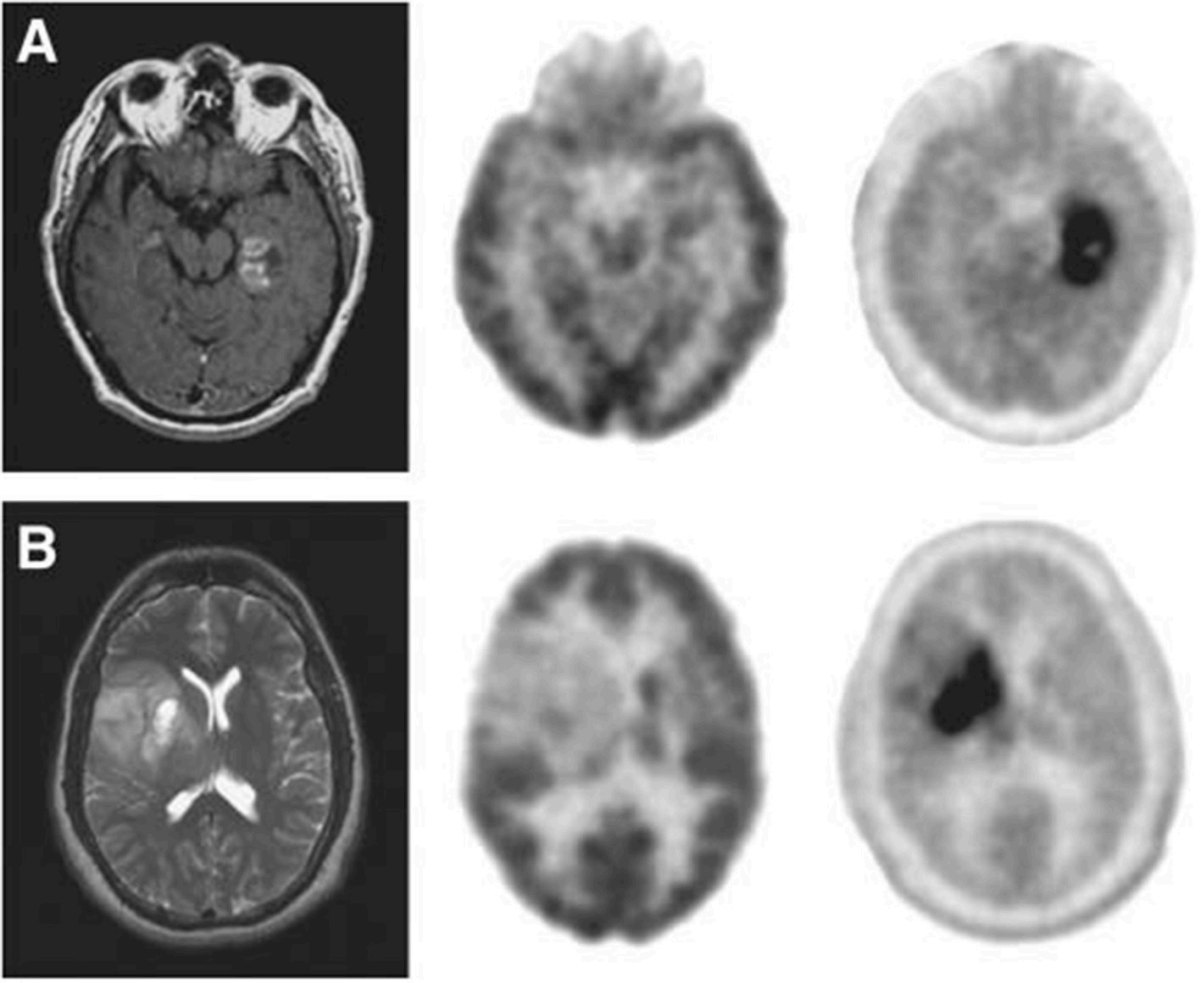
**(A)** Images of a newly diagnosed glioblastoma. **(B)** Images of a newly diagnosed World Health Organization grade II oligodendroglioma. Magnetic resonance (left), ^18^F-FDG PET (middle), and ^18^F-FDOPA PET (right). ^18^F-FDOPA PET imaging illustrated significantly better visualized and defined tumor with adequate contrast. This figure is reproduced with permission from Chen et al. (2006), Figure 2 © by the Society of Journal of Nuclear Medicine Imaging, Inc.

^18^F-FDOPA is an analog of L-dopa, and ^18^F-OMFD is a major metabolite of ^18^F-FDOPA (Beuthien-Baumann et al., 2003; Gulyas and Halldin, 2012). ^18^F-FDOPA has been used to investigate the activity of aromatic L-AA decarboxylase and to evaluate the dopaminergic system functioning in brain tumors and neuroendocrine tumors. ^18^F-FDOPA has been used for detecting primary, metastatic and recurrent brain tumors, and provides valuable information on the delineation of tumor volume, the determination of proliferative activities and grading (Figure 5) (Chen et al., 2006; Fueger et al., 2010; Pafundi et al., 2013; Juhász et al., 2014). The uptake of ^18^F-FDOPA correlates with the glioma grade, thus it plays an important role for managing patients in clinical settings (Fueger et al., 2010; Walter et al., 2012; Pafundi et al., 2013).

There are several AA PET tracers of imaging glutaminolysis, such as L-[5-^11^C]-glutamine (Qu et al., 2012) 4-^18^F-(2S,4R)-fluoroglutamine (^18^F-FGln) (Lieberman et al., 2011), and (2S,4S)-4-(3-^18^F-fluoro-propyl)glutamine (^18^F-FPGln) (Lewis et al., 2015). Study showed that high uptake of ^18^F-FGln in glioma, and ^18^F-FGln may be a helpful tracer for glioma imaging (Venneti et al., 2015).

### Neuroendocrine tumors

Neuroendocrine tumors (NETs) are a heterogeneous group of neoplasms from cells of the endocrine and nervous systems. Identifying the accurate location of primary tumors and metastases are essential for the treatment of NETs. ^18^F-FDG is a nonspecific tracer for NETs, and its uptake is always low in well-differentiated NETs (Huang and McConathy, 2013b).

Knowledge about NETs uptake amine precursors led to the development of ^11^C-HTP and ^18^F-FDOPA.^11^C-HTP is useful for detecting small tumors and early recurrences, however, the 20-min half-life of ^11^C limits the wide clinical use of ^11^C-HTP (Oberg and Castellano, 2011; Toumpanakis et al., 2014).

**Figure 6:**
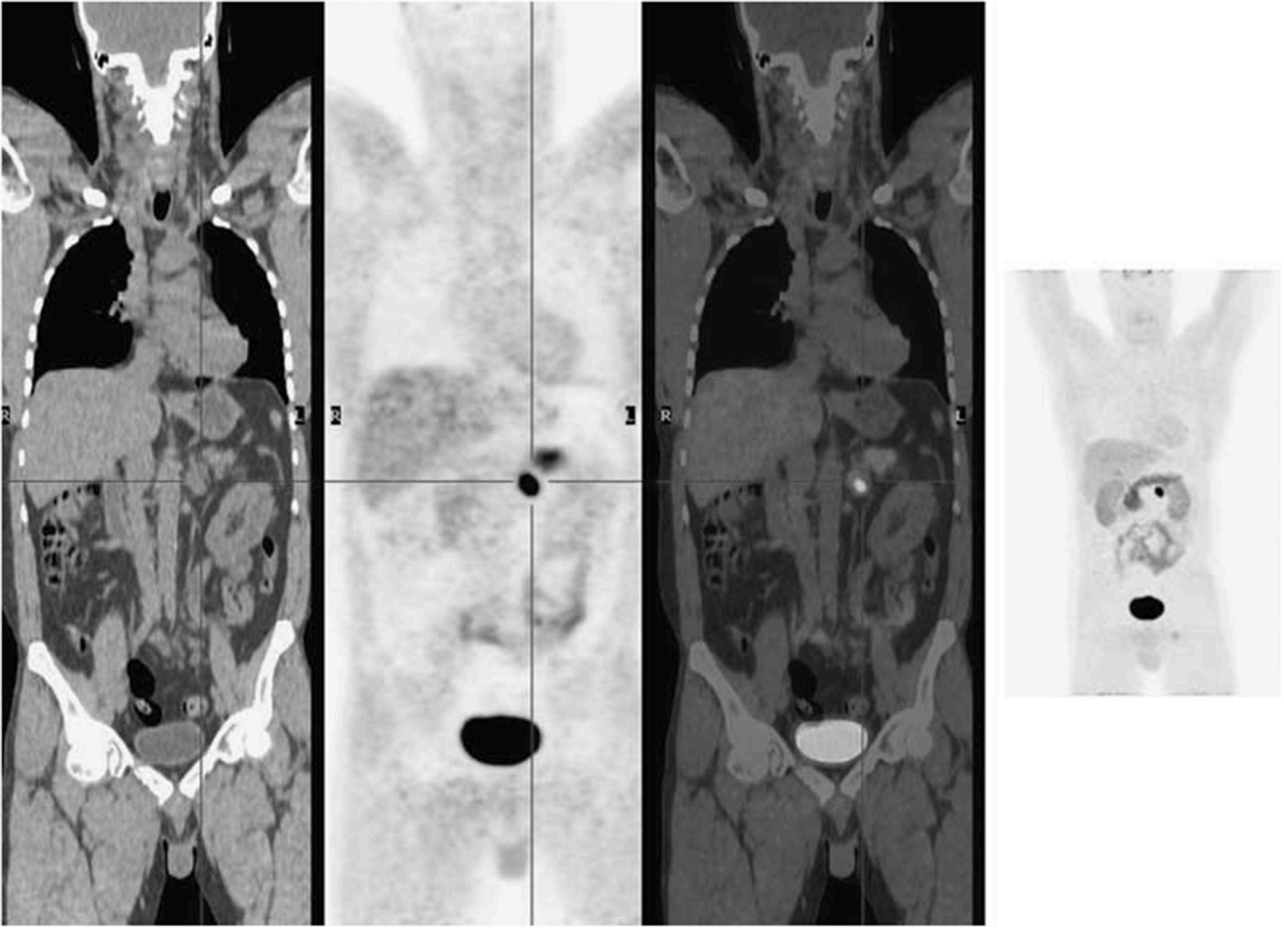
^18^F-FDOPA PET imaging illustrated a solitary left phaeochromocytoma. Left, coronal CT image; mid left, coronal PET image; mid right, coronal fused PET/CT image; right, maximum intensity projection image. This figure is reproduced with permission from Wong et al. (2011), Figure 9 © by the Society of Journal of Nuclear Medicine Imaging, Inc.

NETs increase activity of L-DOPA decarboxylase, so they show a high accumulation of ^18^FDOPA (Jager et al., 2008). ^18^F-FDOPA is a favorable AA tracer for diagnosing NETs with high accuracy, such as pheochromocytomas (Figure 6) (Wong et al., 2011), pancreatic pheochromocytoma and insulinomas, and for staging carcinoids (Koopmans et al., 2006; Timmers et al., 2007; Huang and McConathy, 2013b). Additionally, ^18^F-FDOPA is a highly sensitive marker in patients with functional carcinoid tumors and has low sensitivity for malignant NETs, such as medullary thyroid cancer and pancreatic islet cell tumors (Weisbrod et al., 2012).

One study compared ^11^C-HTP PET and ^18^F-FDOPA PET in patients with gastrointestinal-NET and pancreatic-NET. ^18^F-FDOPA was found to be more sensitive than ^11^C-HTP (98 vs. 89%, respectively) for gastrointestinal-NET. However, for pancreatic-NET, the result was opposite (80 vs. 96%, respectively) (Orlefors et al., 1998; Toumpanakis et al., 2014).

### Prostate cancers

Prostate cancer is a complex and biologically heterogeneous tumor, which is the second leading cause of cancer-related death in the United States and Europe (Huang and McConathy, 2013b). ^18^F-FDG is not an adequate tracer for differentiating prostate cancer, benign hyperplasia lesion and normal prostate (Picchio et al., 2015), and it is not useful for initial staging and is of limited utility in the clinical setting of biochemical failure after prior definitive therapy for primary cancer (Jadvar, 2016). ^11^C-MET is a helpful tracer for imaging the prostate in patients with increased PSA levels (Toth et al., 2005; Jana and Blaufox, 2006). Short dynamic scanning limits the wide clinical use of ^11^C-MET for imaging prostate cancer.

**Figure 7:**
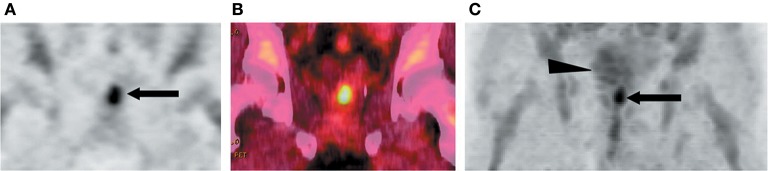
^18^F-FACBC PET images of a 71-year-old man with biopsy-proven prostate bed recurrence. **(A)** Coronal PET and **(B)** coronal fused PET/CT image illustrated the recurrent tumor extending toward left seminal vesicle (arrow in A). **(C)** Maximum-intensity-projection image at 20 min illustrated high uptake in prostate bed (arrow) with little bladder excretion (arrowhead). This figure is reproduced with permission from Schuster et al. (2007), Figure 4 © by the Society of Journal of Nuclear Medicine, Inc.

^18^F-FACBC, an L-leucine analog, is a valuable tracer in the assessment of prostate cancer. Due to its low urinary excretion after injection (Figure 7), it has advantages in the imaging of prostate cancer (Schuster et al., 2007, 2011; Huang and McConathy, 2013b; Picchio et al., 2015). Prostate cancer, within the prostate or in pelvic lymph node metastases, can be detected using ^18^F-FACBC with high sensitivity and specificity (Schuster et al., 2011; Castellucci and Jadvar, 2012). The *vitro* uptake studies demonstrate that ^18^F-FACBC is transported by LAT1 and ASCT2 in prostate cancer cell lines (Oka et al., 2012). More studies are needed to evaluate this radiotracer in the clinical management of men with prostate cancer (Schuster et al., 2011). ^18^F-FACPC, as an analog of ^18^F-FACBC, is a helpful tracer for imaging prostate cancer, but ^18^F-FACPC is not a good tracer for imaging pelvic lymph node metastases compared to ^18^F-FACBC (Savir-Baruch et al., 2011).

### Other tumors

In maxillofacial tumors, the sensitivity of ^18^F-FAMT is higher than that of ^18^F-FDG, demonstrating that the accurate diagnosis of maxillofacial tumors is possible with ^18^F-FAMT (Miyakubo et al., 2007).

Head and neck cancer can be imaged with ^11^C-MeAIB. ^11^C-MeAIB shows active and rapid transport into tumor tissues and salivary glands (Sutinen et al., 2003). ^11^C-MeAIB is also helpful in the differential diagnosis of pulmonary and mediastinal mass lesions (Nishii et al., 2013). ^18^F-D-FMT (BAY 86-9596), a derivative of ^18^F-labeled tyrosine and is transported via the system L transporter 1 (LAT-1), showed a lower sensitivity but higher specificity for ^18^F-D-FMT than ^18^F-FDG in patients with NSCLC and head and neck squamous cell cancer and (Burger et al., 2014).

4-borono-2-^18^F-fluoro-phenylalanine (^18^F-FBPA) was developed to predict ^10^B concentrations, presumably after administration of boron-containing drug for neutron-capture therapy (BNCT) (Wang et al., 2004; Menichetti et al., 2009; Tani et al., 2014). Studies showed that ^18^F-FBPA, was transported by system L, could evaluate BPA uptake in tumors for screening candidates for BNCT (Havu-Auren et al., 2007; Menichetti et al., 2009; Yoshimoto et al., 2013). However, the inconsistent result was showed that ^18^F-FDG might be an effective tracer prior to ^18^F-FBPA for screening patients with head and neck cancer for treatment with BNCT (Tani et al., 2014; Kobayashi et al., 2016).

## Conclusion and prospects

AA PET tracers can overcome the shortcomings of ^18^F-FDG and provide more information for imaging tumors. Uptake mechanism of AA PET tracers involves protein synthesis or AA transport. For PET imaging, AA transport tracers appear more valuable than protein synthesis tracers in clinical applications. Targeting AA transporter system A, ASC, L and X_C_−, have been used in the clinical imaging of the biological behaviors of various tumors. Transporter system L has been a major focus of tracer development for imaging tumors (such as ^11^C-MET, ^18^F-FET, ^18^F-FDOPA) and has also led to several AA tracers that are effective for imaging neuroendocrine tumors (^18^F-FDOPA) and prostate cancer (^18^F-FACBC) (Huang and McConathy, 2013a). ^18^F-FSPG (BAY 94-9392), which is specific for system X_C_− transporters (Koglin et al., 2011; Smolarz et al., 2013a), has been used for imaging patients with hepatocellular carcinoma (Baek et al., 2013), NSCLC (Smolarz et al., 2013a) and breast cancer (Chopra, 2004; Baek et al., 2012). Recently, new ^18^F-labeled branched-chain AAs have been developed that target cationic AA transporter and excitatory AA transporters X_AG_−, which are potential targets of AA PET tracers for tumor imaging. O-2((2-[(18)F]fluoroethyl)methylamino)ethyltyrosine (^18^F-FMAET) is specific for cationic AA transporter (Chiotellis et al., 2014). BAY 85-8050, a glutamate derivative, is specific for transport system X_C_− and systems ${\text{X}}_{\text{A}{\text{G}}^{-}}$, which is used to study healthy volunteers (Krasikova et al., 2011; Ploessl et al., 2012).

Besides branched-chain AAs, novel *N*-substituted labeled AAs and AA mimetics, have also been developed. ^18^F-FPGLU is *N*-methylsubstitutebeled amino glutamic acid as a potential AA tracer for PET imaging of transporter X_AG_− and X_C_− in tumor, and can be used for clinical tumor imaging in the near future. ^18^F-Phe-BF_3_ (an exotic replacement of the carboxylate with -BF_3_) is a new class of AA mimetics-boramino acid tracer for PET imaging of transporter LAT1 in tumor, with specific accumulation in U87MG xenografts and low uptake in normal brain and an inflammatory region (Liu et al., 2015). Also, synthesis of novel AAs with conformationally constrained side chains will lead to developing a series of new radiolabeled AA mimetics for imaging disease, with good prospect (Mollica et al., 2010, 2012; Stefanucci et al., 2011; Way et al., 2014).

Novel radiolabeling techniques are developing for radiosynthesis of AA PET tracers, resulting in routine high-dose production of AA tracers for clinical PET imaging. Recently, the no-carrier-added (NCA) enantioselective synthesis using a chiral phase-transfer catalyst has been used for automated synthesis of NCA ^18^F-FDOPA with the Curie Level (Libert et al., 2013), and simple and efficient two-step synthesis of ^18^F-FDOPA with short synthesis times can supply adequate radioactivity for clinical imaging (Tredwell et al., 2014). Thus, ^18^F-FDOPA is easily available and will become widely used AA PET tracer for the detection of brain tumors, neuroendocrine tumors, Parkinson's disease (PD), and mental illness (Darcourt et al., 2014; Eggers et al., 2014; Li et al., 2014). Simple and practical click reaction and ^68^Ga labeling methods are also used for preparing new AA tracers for imaging tumors, which will further boost translational application of AA tracers in clinics.

## Author contributions

GT is the corresponding author for summarize amino acids PET tracers and the future about amino acids PET. He also reviewed this paper. AS searched literature and wrote the manuscript. XL searched literature and drew the figure and table.

### Conflict of interest statement

The authors declare that the research was conducted in the absence of any commercial or financial relationships that could be construed as a potential conflict of interest.
